# Real-time GIS data model and sensor web service platform for environmental data management

**DOI:** 10.1186/1476-072X-14-2

**Published:** 2015-01-09

**Authors:** Jianya Gong, Jing Geng, Zeqiang Chen

**Affiliations:** State Key Laboratory of Information Engineering in Surveying, Mapping and Remote Sensing, Wuhan University, 129 Luoyu Road, Wuhan, 430079 China; Collaborative Innovation Center of Geospatial Technology, 129 Luoyu Road, Wuhan, 430079 China

**Keywords:** Real-time GIS data model, Sensor Web service platform, Environmental data management

## Abstract

**Background:**

Effective environmental data management is meaningful for human health. In the past, environmental data management involved developing a specific environmental data management system, but this method often lacks real-time data retrieving and sharing/interoperating capability. With the development of information technology, a Geospatial Service Web method is proposed that can be employed for environmental data management. The purpose of this study is to determine a method to realize environmental data management under the Geospatial Service Web framework.

**Methods:**

A real-time GIS (Geographic Information System) data model and a Sensor Web service platform to realize environmental data management under the Geospatial Service Web framework are proposed in this study. The real-time GIS data model manages real-time data. The Sensor Web service platform is applied to support the realization of the real-time GIS data model based on the Sensor Web technologies.

**Results:**

To support the realization of the proposed real-time GIS data model, a Sensor Web service platform is implemented. Real-time environmental data, such as meteorological data, air quality data, soil moisture data, soil temperature data, and landslide data, are managed in the Sensor Web service platform. In addition, two use cases of real-time air quality monitoring and real-time soil moisture monitoring based on the real-time GIS data model in the Sensor Web service platform are realized and demonstrated. The total time efficiency of the two experiments is 3.7 s and 9.2 s.

**Conclusions:**

The experimental results show that the method integrating real-time GIS data model and Sensor Web Service Platform is an effective way to manage environmental data under the Geospatial Service Web framework.

## Background

Environmental data are some of the most critical information sources for evaluating, preventing, and alleviating the adverse effects of the environment on human health. Effective environmental data management plays an important role in retrieving and applying environmental data. In the past, environmental data management involved developing a particular application and isolated environmental data management system [1–5]. With the development of sensor technology, sensors have become smaller, cheaper, more intelligent, and more power-efficient [6]. A large number of sensors are deployed for environmental monitoring, and plenty of real-time spatiotemporal environmental data are generated. Static geographic information handling extends to dynamic real-time data handling [7], and environmental data management systems heavily rely on integrating and consolidating heterogeneous sensor data streams [8]. However, many of the particular application and isolated environmental data management systems cannot meet the requirements of managing real-time data.

Recently, with the development of information technologies such as Web services and interoperable services, a Geospatial Service Web (GSW) has been proposed in the geospatial community. GSW is a virtual geospatial infrastructure based on the Internet, and it integrates various geospatial-related resources such as sensor resources, data resources, processing resources, information resources, knowledge resources, computing resources, network resources, and storage resources to manage data, extract information, and obtain knowledge in the geospatial community domain [7]. GSW unifies the functions of a geospatial acquisition system, data transformation system, distributed spatial data collection, high-capability server system, large volume storage system, remote sensing, and a geographic information system (GIS), where the functions are implemented by Web services and communicated through the standardized protocols of the Internet. The mission of GSW includes the following: 1) acquire global spatial data for all seasons, all days, and all directions using all kinds of sensors on satellite, aircraft, and ground surface; 2) chain the whole process seamlessly from sensors to application services using unified information networks, including satellite communication, data relay network, and wired or wireless computer communication networks; 3) register sensors, computing resources, storage resources, internet resources, manipulate software and spatial data on the internet, and process spatial data online quantitatively, automatically, intelligently, and in real time; and 4) provide geospatial services, compose virtual service chains and transmit user-required information in the most effective and efficient ways. Using GSW for real-time environmental data manage will help describe, organize, manage, manipulate, interchange, search, and release environmental data in a unified framework.

Currently, the GSW is a conceptual framework. It will be a long-term task to realize the blueprint [7]. An urgent task for GSW is developing a real-time GIS data model to manage real-time data. To date, GIS data models have evolved from static GIS data models, to temporal GIS data models, and then to real-time GIS data models [9]. A static GIS data model manages spatial data, describes spatial relationships, and expresses the distributions of geospatial objects. Based on the static GIS data model, a temporal GIS data model adds the description information of time. The temporal GIS data model represents the distributions of geographic objects and the change process of these objects with time. The temporal GIS data model can be divided into three phases according to the significance of time in a model: 1) temporal snapshots phase: focusing on recording an entity’s snapshots in their temporal changes. Typical data models include the space-time cube model [10, 11], sequential snapshots model [12, 13], discrete grid cell list model [14, 15], base state with amendments [13, 15], and the space-time composite model [16–18]. This type of data model is primarily used for recording the state changes of the entity itself with time to store and retrieve spatial characteristics and special features of an object. 2) object change phase: focusing on the changed relationship of an object before and after its change. Some famous data models include the object-oriented spatiotemporal data model [19–21], feature-based spatiotemporal date model [22], and process-oriented spatiotemporal data model [23, 24]. The data models record the change states of the entity itself and also describe the changed relationship of the states to express the spatiotemporal changed relationship between geographic objects. 3) events and action phase: focusing on describing the semantic relations of an entity’s changes. Some well-known data models include the event-based spatiotemporal data model [13], graph-based spatiotemporal data model [25], and spatiotemporal three-domain model [18]. Compared with the data models in the object change phase, a benefit of the data models in the events and action phase is implying the reason for the spatiotemporal change of a geographic object state. This helps to express the interactive relationship between geographic objects, or a geographic object with an external environment. The temporal GIS data models are primarily used to express the geographic object changes through time, store masses of history data, and maintain their relationships. However, most of them are employed to represent the object changing from one kind of state to another. They are often not effective for storing and retrieving real-time spatial data from various sensors and moving objects, lacking of real-time capability to meet the increasing demand for time-sensitive applications. The real-time GIS data model is developed from the temporal GIS data model and emphasizes the time efficiency of data management [26, 27]. Currently, the real-time GIS data model is still in an immature stage and needs further study.

The objective of this study is to propose a method to manage real-time environmental data. This method is based on a novel real-time GIS data model and the model’s implementation called the Sensor Web Service Platform with Sensor Web technologies [6]. The proposed real-time GIS data model in this study collects real-time data from various types of sensors and represents the relationships between data such as geographic objects, states, events, processes, sensors, and observations. The model also supports the dynamic simulation of spatiotemporal processes from real-time GIS data. The real-time GIS data model represents further progress for static and temporal GIS data models.

## Methods

This section demonstrates the proposed real-time GIS data model and one method of implementation, called the Sensor Web Service Platform.

### Real-time GIS data model

The real-time GIS data model is actually a spatiotemporal data model for real-time GIS. Real-time GIS is an important new research domain, transforming the study of historical changed data to real-time data in GIS [28]. Compared with traditional GIS, real-time GIS has strict time controls and restraints; therefore, all actions will be performed in a very short and acceptable time. The data model is the core of GIS; an appropriate data model plays a decisive role in constructing a GIS application. The primary task of a spatiotemporal data model is the organization and management of spatiotemporal data, as well as analysis and expression of the content and relationships of spatiotemporal change. This study draws lessons from existing spatiotemporal data models, analyses the evolutive mechanism of a spatiotemporal process, studies the retrieval of real-time observation data derived from sensors and mobile targets, and then develops a real-time GIS data model.

**Figure 1:**
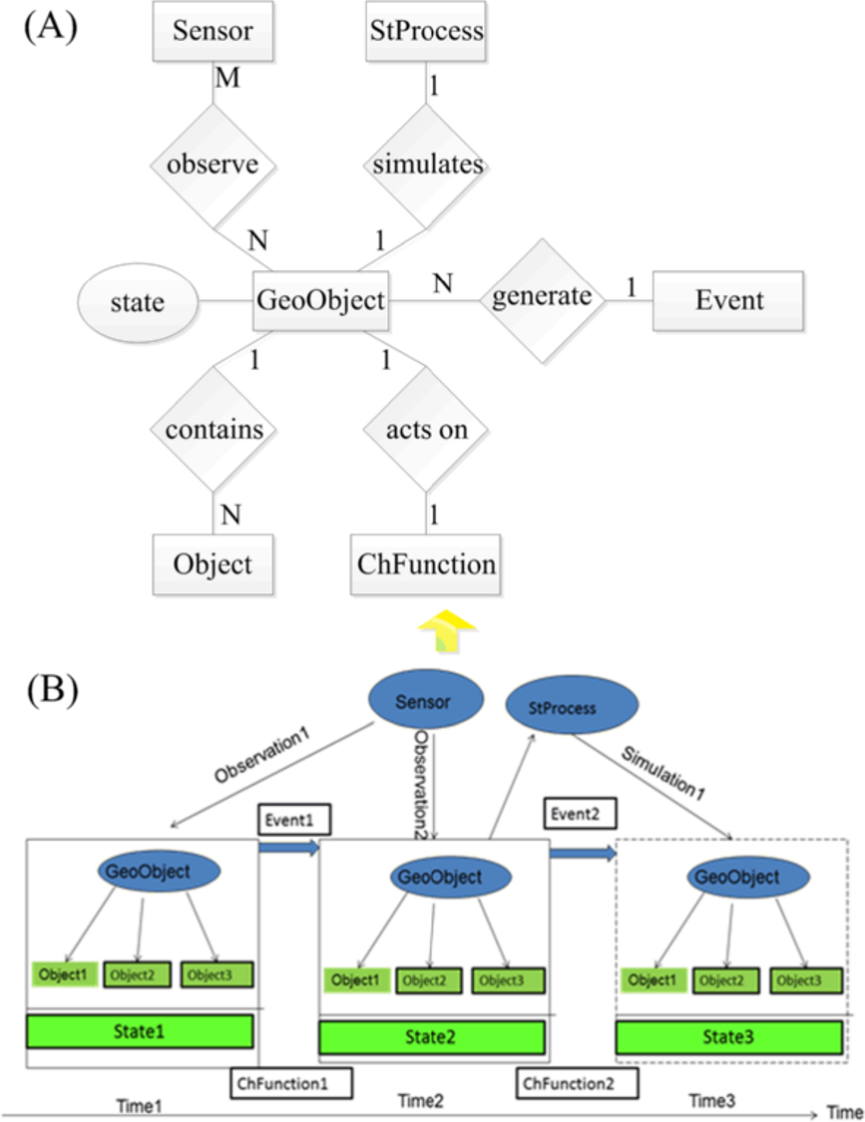
**The real-time GIS data model. (A)** is the Entity-Relationship diagram; **(B)** is the conception diagram.

Spatiotemporal variation is an eternal theme of the objective world; feature entities and phenomena always change quickly or slowly with time. A complex geographic phenomenon often refers to a number of geographic objects. The geographic objects and their interactions control the change of the complex geographic phenomenon. Action of a geographic phenomenon that occurred at a time point is an event. An event will occur when the change of an object reaches a certain degree. Under certain conditions, an event drives the corresponding change of geographic objects, and the change in the geographical objects is recorded via a sequence of object states. The whole process of change over time and space forms the geographical spatiotemporal process. To understand the temporal and spatial change, a geographic object's attribute data should be derived directly from real-time observation by a sensor. For example, air quality changes can be monitored in a certain place over a certain time period. Air quality is a geographic phenomenon that depends on a variety of air pollutants such as carbon monoxide or fine particulate matter. When the concentrations of some air pollutants reach a certain degree, an air pollution event occurs. According to the pollution degree, several pollution states are determined, e.g., mild contamination and serious contamination. The changes in air quality in the place during the time period are a spatiotemporal process. People monitor the concentration of air pollutants using sensors to perform quantitative analysis of contamination. Based on these analyses, a real-time GIS data model is proposed to store and manage the spatiotemporal data involved in the spatiotemporal change process of a phenomenon to support applications of real-time GIS visualization and analysis. The data model should have five characteristics: 1) the model takes into account both traditional GIS and real-time GIS; 2) it can express the dynamic data from moving object; 3) it is highly effective for storing and retrieving real-time data from various sensors; 4) it can support the dynamic simulation of spatiotemporal processes from real-time GIS data; and 5) the model can represent the relationships among its factors, including geographic objects, states, events, processes, sensors and observations. The real-time GIS data model is depicted in Figure 1. Figure 1(A) is the Entity-Relationship diagram [29] and Figure 1(B) is the conception diagram. Some relevant elements of the conceptual model are described as follows:

- **Sensor (Sensor)**: Various sensors containing space-borne, air-borne, and ground sensors.
- **Observation (Observation)**: The behaviour of observable attributes from various sensors provides observational data for the model.
- **Geographic Object (Geo-Object)**: Either physical entities or social phenomenon formed naturally or artificially, expressed with clear boundaries or not, as the objects of GIS research in the real world.
- **Object (Object)**: Single entity in the real world; a Geo-Object can contain one or multiple objects.
- **Spatiotemporal Process (StProcess)**: The Spatiotemporal Process is a periodized change process of a complex geographic phenomenon in a timeline, and the processes refer to a series of Geo-Objects and their interactions.
- **Simulation (Simulation)**: Simulation is the imitation of the operation of a real-world process or system over time.
- **Event (Event)**: An event is an occurrence of the Geo-Object change, and is the reason for the change of Geo-Objects.
- **State (State)**: A snapshot of a geographic object at a point of time in the change process.
- **Change Function (ChFunction)**: In the time of research, the correspondence between an instant and the values of geospatial and thematic properties. This function can be derived from industry, scientific computing, and relevant experience.

A geographic object consists of three basic indivisible features: time, space, and thematic attributes [15, 30]. A geographic object contains both unchangeable attributes and time-varying attributes. Time-varying attributes are associated with state sequences. The time-varying attributes may be different at different states.

A sensor is a special geo-object that contains self-parameters and observations. The sensor, described by its metadata, is a tool to observe the spatial attributes and the thematic attributes of geographic objects; therefore, a sensor is the primary means of obtaining the changed information of a geographic object. One sensor may observe many geo-objects; meanwhile, a geo-object can be observed by many sensors. The wide use of sensors has brought revolutionary changes to data acquisition by improving the accuracy, speed, timely perception, and timely transmission of spatiotemporal data. This change has resulted in the generation of a large volume of data, such as spatiotemporal data, thematic attribute data, image data, and video stream data. This information, which may be remote sensing image collected by a remote sensor, physical or chemistry parameters collected by an in-situ sensor, or only position information acquired by a Global Navigation Satellite System, is recorded in a series of observations along with the time.

Complex spatiotemporal changes in geographical phenomena refer to three core things: spatiotemporal processes, geographic objects, and events. A real-time GIS data model should not only be able to express and manage real-time sensor observation data, but also should express and manage spatiotemporal process. To support the spatiotemporal process, it should reveal the relationships between geographic objects, events, and spatiotemporal processes. An event is an occurrence whereby a geo-object changes in a spatiotemporal process [13, 31]. A geo-object generates an event, and an event drives the change of a geo-object from one state to another with the change function. If the relationships between the geo-object, event, function, and spatiotemporal process are known, the state information can also be simulated by spatial-temporal processes with events with respect to the prior state and change function. The data model must handle all elements and establish their relationships, as in Figure 1.

### Sensor web service platform

A Sensor Web can obtain, access, manage, and process sensor data in a standardized way in real-time or near real-time [32–35]. Therefore, a Sensor Web Service Platform integrating Sensor Web technologies to provide the interfaces in GSW for registering, planning, and monitoring various space-borne, airborne, and ground sensors is adopted to support the realization of the real-time GIS data model.

The Sensor Web is an infrastructure providing a bridge between sensor resources (sensors and sensor systems) and their applications, where the infrastructure enables an interoperable usage of sensor resources by enabling their discovery, access, and tasking, as well as eventing and alerting in a standardized way [6]. The Open Geospatial Consortium (OGC) Sensor Web Enablement defined the Sensor Web information model and interface model. The information model defines the encoding standards of sensor observations and sensor metadata, such as the Observations & Measurement [36] and the Sensor Model Languages (SensorML) [37]. The interface model specifies the interfaces of the different Sensor Web services such as the Sensor Observation Service (SOS) [38], the Sensor Planning Service (SPS) [39], and the Sensor Event Service (SES) [40]. The SOS provides a standardized interface to manage and retrieve metadata and observations from heterogeneous sensor systems. The SPS defines interfaces for queries that provide information about the capabilities of a sensor and how to task the sensor. The SES is an enhancement of the OGC Sensor Alert Service, and it provides operations to register sensors at the service application and let clients subscribe to observations available at the service.

**Figure 2:**
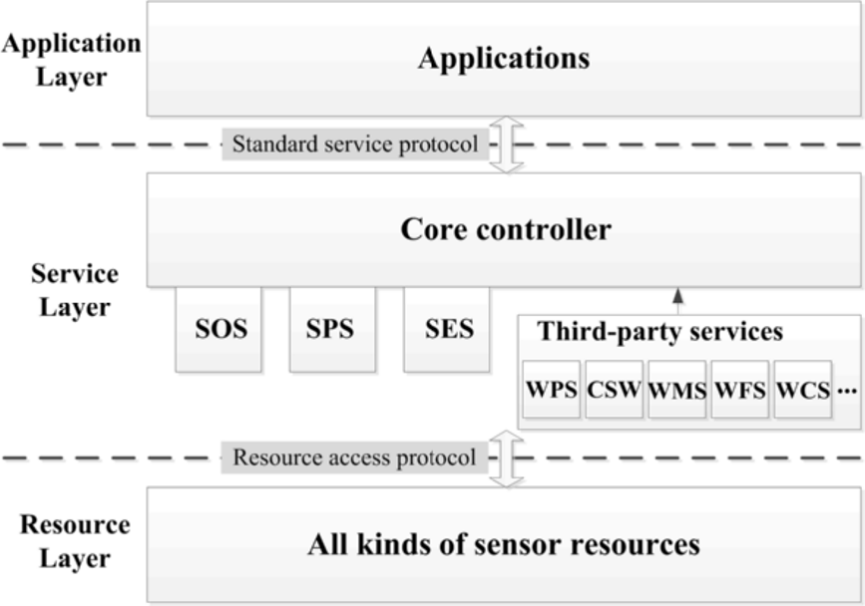
**The framework of the Sensor Web Service Platform.**

The Sensor Web Service Platform follows the layer-based framework given in Figure 2. There are three layers in the framework: a resource layer, service layer, and application layer. The service layer not only provides the standard Sensor Web services (SOS, SPS, and SES), but also enables integrating third-party services such as the commonly used OGC services Web Processing Service [41], Catalog Service for the Web, Web Map Service, Web Feature Service, and Web Coverage Service, using a core controller component. The service layer interacts with the resource layer and the application layer using a resource access protocol and standard service protocol, respectively.

## Results

To demonstrate the proposed method for environmental data management, a Sensor Web Service Platform was implemented that supported the realization of the real-time GIS data model. Two cases of environmental data management are shown for Wuhan city, China. One is real-time air quality monitoring, and the other is real-time soil moisture monitoring.

### A prototype of sensor web service platform

**Figure 3:**
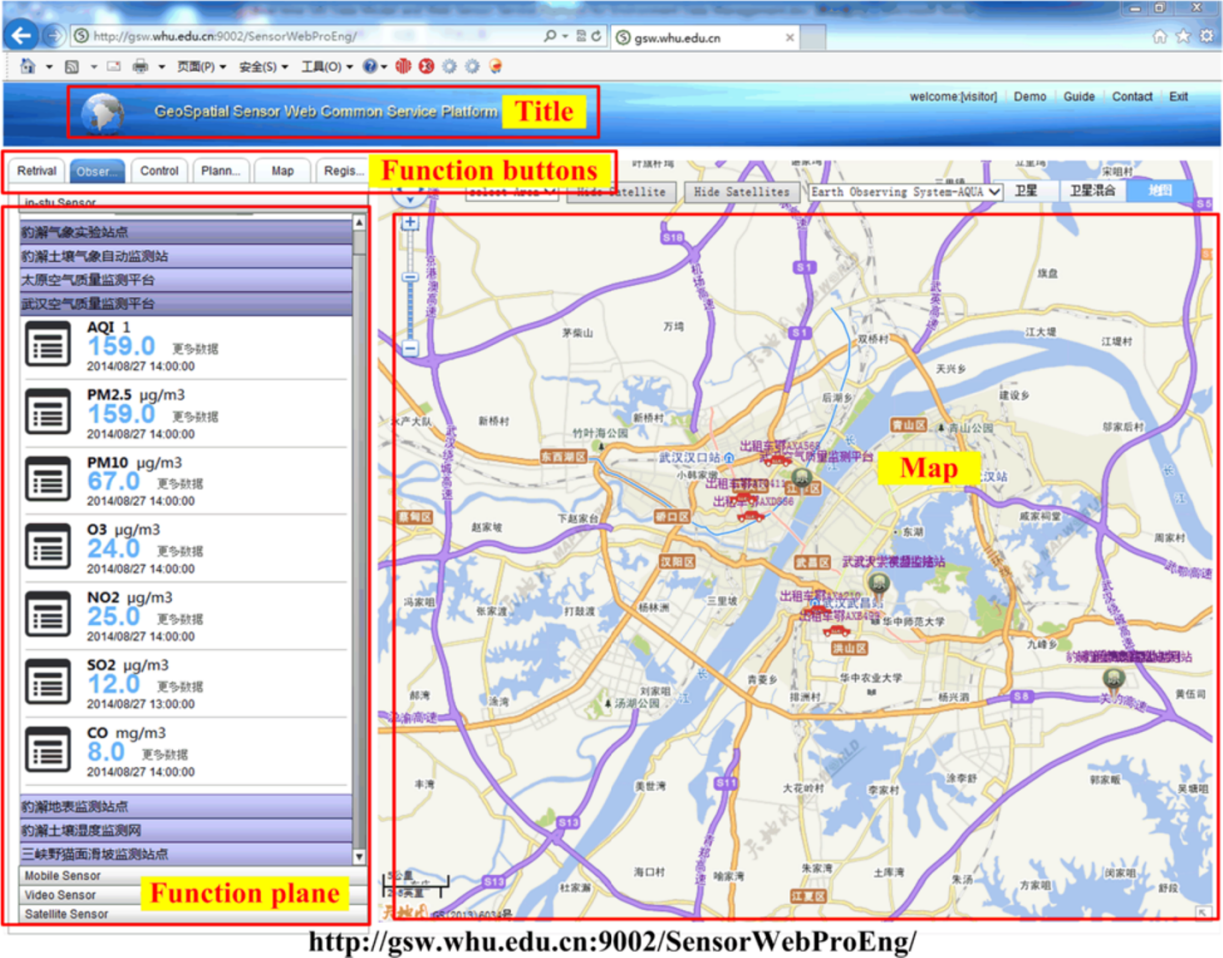
**The Web portal of the Sensor Web Service Platform.**

A Sensor Web Service Platform (its Web portal as Figure 3) consistent with the framework of Figure 2 was implemented with Sensor Web technologies by the Sensor Web group of Wuhan University, China [42, 43]. The experiments in this study are based on the Sensor Web Service Platform and follow the notion of the real-time GIS data model. The objective of the Sensor Web Service platform is to provide an integrating environment for sensor resources under the GSW framework. The Sensor Web Service Platform integrates sensor registration service, sensor observation service, sensor planning service, real-time mapping service, satellite positioning service, and other services to obtain real-time sensor information, observational data, data products, and other information resources; it also vividly demonstrates these information resources in the Map World [44] with graphics, text, tables, and video. The Sensor Web Service Platform primarily consists of six major functional modules:

- Sensor Retrieval Module: retrieves motion sensors, in-situ sensors, and remote sensors according to the specified filter criteria, such as time, space, subject, and other constraints;
- Sensor Observational Data Retrieval Module: provides access to various types of sensor observation data according to the specified filter conditions and then shows these observational data in Map World in different ways;
- Sensor Control Module: controls in-situ sensors and video sensors, and provides feedback for sensor control based on these changes in sensor observational data or the method of accessing sensor observational data;
- Sensor Planning Module: performs video sensor planning tasks and remote sensing satellite simulative planning tasks;
- Thematic Map Module: generates thematic maps with observation data; the maps can reflect the overall situation in a specific area;
- Sensor Registration Module: used to register sensors described by the SensorML format. Sensor Registration Module is used to register sensor described with SensorML format.

Currently, dozens of sensors and plenty of real-time environmental data are managed by the Sensor Web Service Platform with real-time GIS data models, such as meteorological data (wind speed, wind direction, sunshine duration, solar radiation, atmospheric pressure, air temperature, air humidity, rainfall), air quality data (air quality index (AQI), particulate matter smaller than 2.5 μm (PM2.5), respirable suspended particulate matter smaller than 10 μm (PM10), ozone (O3), nitrogen dioxide (NO2), sulfur dioxide (SO2), carbon monoxide (CO)), soil moisture data, soil temperature data, and landslide data.

### Real-time air quality monitoring

With the rapid economic growth and urbanization in Wuhan (the capital city of Hubei province in China), air-pollution events such as fog or haze strike Wuhan many times each year. Air quality affects people’s lives, as well as their health. Governments and citizens pay more attentions to air quality than ever before. A governmental agency named the Wuhan Environmental Monitoring Center has instituted some environmental monitoring stations and deployed many sensors in Wuhan to monitor SO2, NO2, PM10, CO, O3, and PM2.5 pollutants, as well as the air quality.

The AQI, a dimensionless index, is a quantitative description of the air quality status as one indicator to monitor air quality. The United States Environmental Protection Agency has released a guideline for standardizing AQI and individual AQI (IAQI; the AQI of individual pollutants) calculation methods and category descriptors to provide health guidelines for the public [45]. The AQI calculation method is adopted by this study.

**Figure 4:**
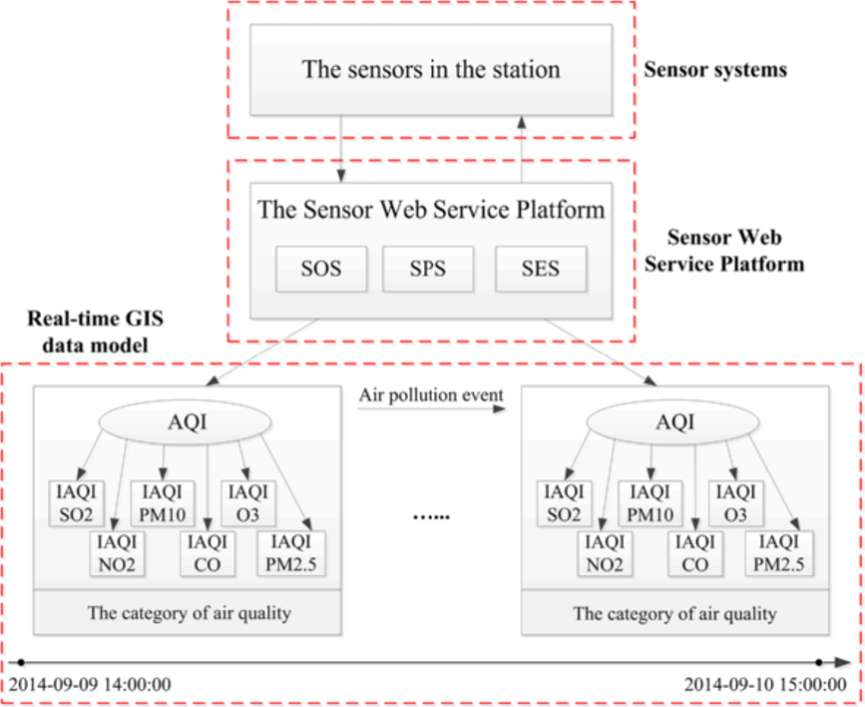
**AQI experiment design.**

To monitor the AQI, a Sensor Web Service Platform and real-time GIS model were designed, as shown in Figure 4.

The real-time GIS data model obtains real-time observation data from the SOS in the Sensor Web Service Platform. In this experiment, the real-time GIS data model parameters are set as in Table 1.

**Table 1:** **The parameters of the real-time GIS model for air quality monitoring**

The real-time GIS data model	Parameter(s) in the experiment
Sensor	The sensors monitor SO2, NO2, PM10, CO, O3, and PM2.5
Observation	The concentration data of SO2, NO2, PM10, CO, O3, and PM2.5 observed by the sensors
Object/Geo-Object	The AQI and the IAQIs of the SO2, NO2, PM10, CO, O3, and PM2.5 in Wuhan.
StProcess	The process of simulating the observation
Event	Air pollution
State	The category of air quality (“Good”, “Moderate”, “Unhealthy for Sensitive Groups”, “Unhealthy”, “Very Unhealthy”, “Hazardous” [25])
Time point	The hours during the period between 2014-09-08 14:00 to 2014-09-10 15:00

The real-time data used in this experiment come from the Wuhan Environmental Monitoring Center. The experimental time period was from 2014-09-08 14:00 to 2014-09-10 15:00. The sensors were registered for monitoring SO2, NO2, PM10, CO, O3, and PM2.5 into SOS, and then the real-time data of the sensors was inserted into SOS by the InsertObservation operation. With the natural real-time characteristics [6], the Sensor Web Service Platform enables the management of real-time observation data. Every hour, the SOS receives live records from the station, and the delay is 1.7 s. All the pollution data are managed by SOS. If any data are required, they are retrieved from SOS with the GetObservation operation. Information or knowledge from the real-time data is mined using the real-time GIS data model.

**Figure 5:**
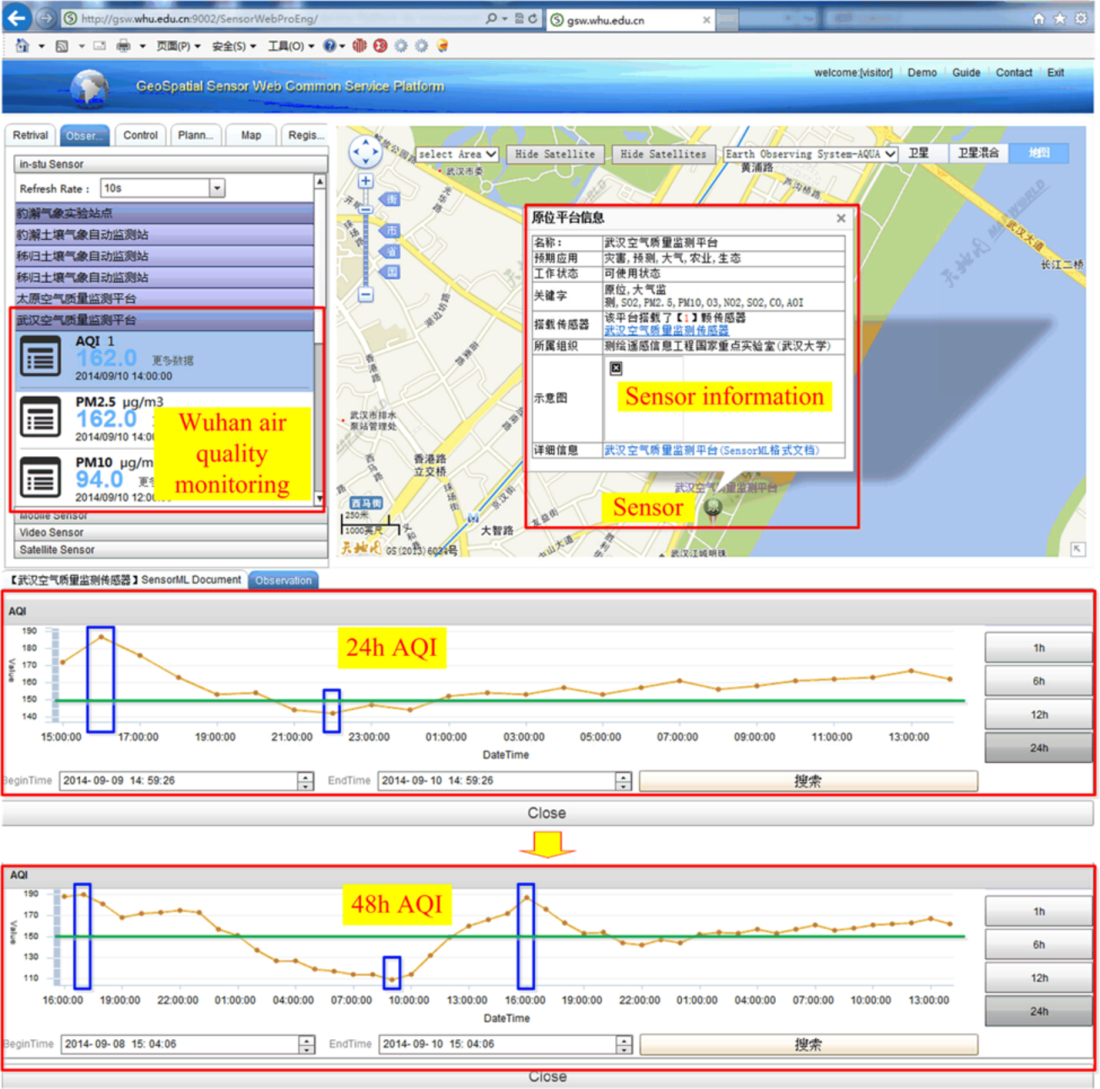
**The average concentrations of pollutants at time points and intervals.**

The real-time GIS data model can show the data at a specific time point and also can exhibit the data series in a time interval. For example, Figure 5 depicts the pollutants at various time points and then the time intervals. The real-time GIS data model obtains the real-time data from SOS with the GetObservation operation. If the request parameter of the GetObservation operation is set at a time point, the response data are singular. If the request parameter is set at a time interval, the response data are serial. The results show that the real-time GIS data model can exhibit real-time data interacting with the SOS in the Sensor Web Service Platform.

Real-time information is mined by the real-time GIS data model. The response time is about 2.0 s. From Figure 5, some observations can be made: 1) during the 24-h experimental period, the highest AQI was observed at 16:00, and the lowest at 22:00. During the 48 hours, the two highest AQI values are at approximately 16:00, while the lowest AQI is at 10:00, which is different from the result of the 24-hour observation. 2) During the 48 hours, the AQI values are between 100 and 200. This corresponds to a rating of “unhealthy for sensitive groups” (AQI from 101 to 150) or “unhealthy” (AQI from 151 to 200) [45]. 3) Comparing the 24-hour AQI data and the 48-hour AQI data, nearly half of the AQI is less than 150 during the first 24 hours, while only one-sixth of the AQI is below 150 during the last 24 hours; thus, the air quality is steadily worsening.

### Real-time soil moisture monitoring

**Figure 6:**
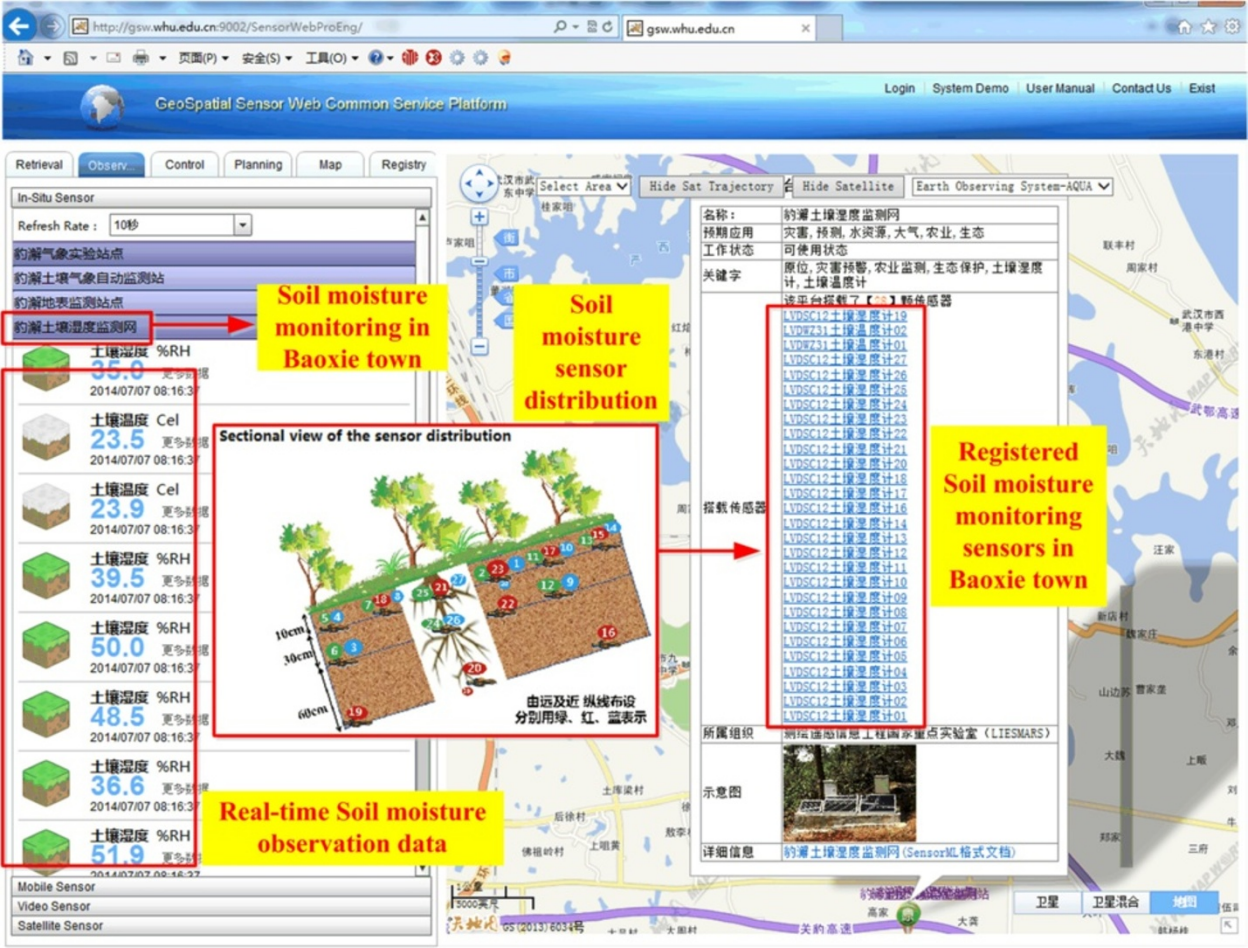
**Implementation of the soil moisture monitoring.**

Soil moisture is an important environmental indicator for studying climate change, reflecting the degree of agricultural drought, and guiding agricultural irrigation. An automatic observation station with more than 20 soil moisture sensors in a 20 m × 40 m experimental area (center location at 114°31′35.61″E 30°28′12.98″N) was constructed at Baoxie town, Wuhan. Sensors were deployed in horizontal planes with three different depths (10 cm, 30 cm, and 60 cm) as the Figure 6. These soil moisture sensors are registered and managed in the Sensor Web Service platform.

In this experiment, the real-time GIS data model parameters are set as in Table 2.

**Table 2:** **The parameters of the real-time GIS model for real-time soil moisture monitoring**

The real-time GIS data model	Parameter(s) in the experiment
Sensor	The soil moisture sensors in Baoxie town
Observation	The values of the soil moisture observed the sensors
Object/Geo-Object	Soil moisture in Baoxie town experiment area
StProcess	The process of simulating the observation
Event	Soil drought
State	The degree of the soil drought
Time point	The hours during the period between 2014-07-05 to 2014-07-07

**Figure 7:**
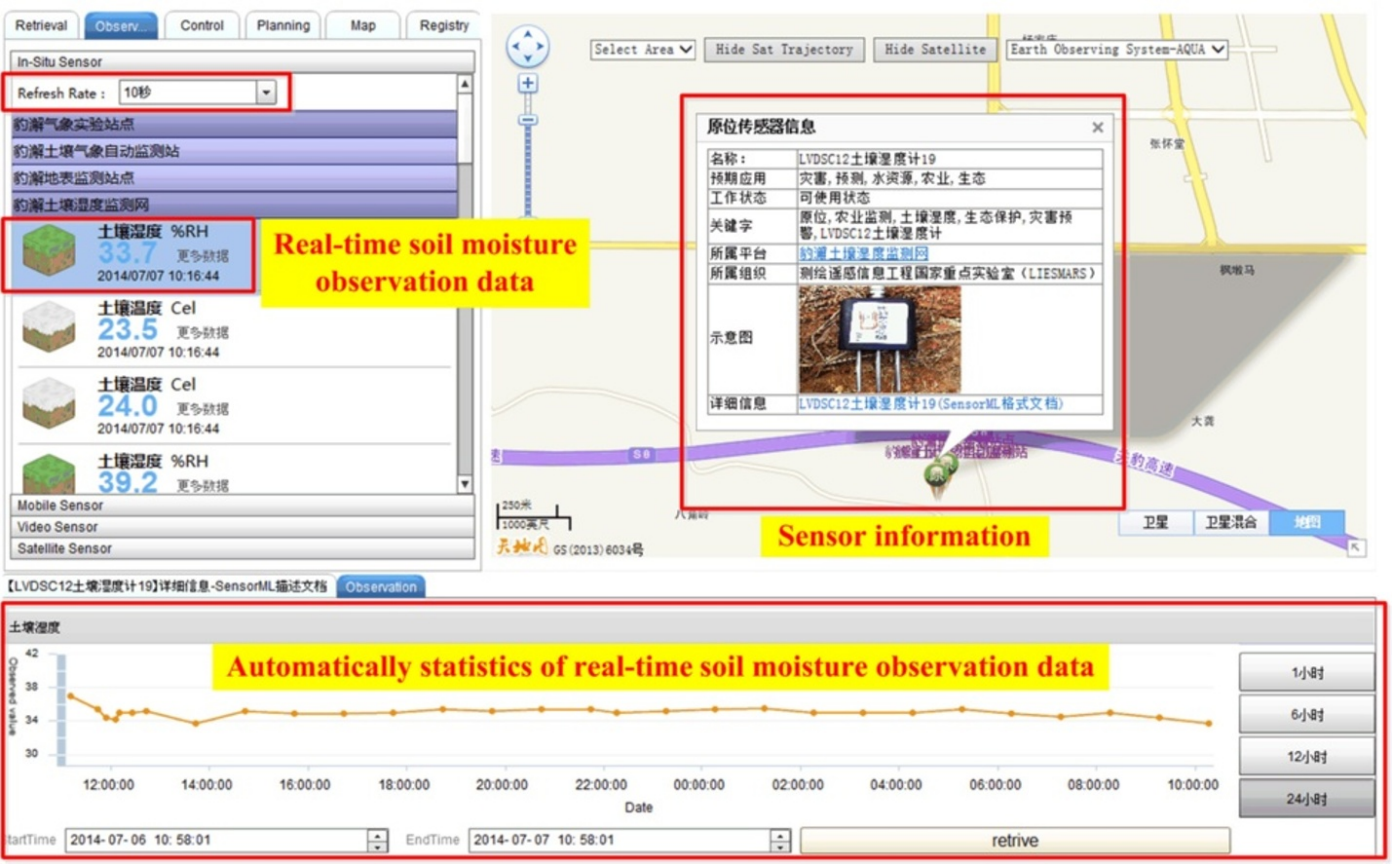
**The Web portal to show real-time soil moisture sensors and their observations.**

The real-time soil moisture was captured by the sensors and then transmitted back through communication channels as GPRS [46, 47]. The time transmitting per record is 1.7 s. The Sensor Web Service Platform manages the observed soil moisture with SOS and visualizes the soil moisture on the Web portal as in Figure 7. SPS controls the sensor sampling time. In the Web portal, the soil moisture sensor information and soil moisture value are listed. For a sensor, during a set time period, the soil moisture can be automatically plotted as a curve, see Figure 7.

Monitoring the soil moisture conditions in the whole experimental area will enable the determination of the drought degree in the area. The deployed soil moisture sensors only monitor discrete points in the area and their number is limited; thus, an interpolation method is needed to find the soil moisture value at every unobserved point according to nearby observed points. Inverse Distance Weighted Interpolation (IDWI), one of the most frequently used spatial interpolation methods, is relatively fast, easy to compute, and straightforward to interpret [48]. In the soil community, IDWI has been used for soil fertility maps [49, 50]. For these reasons, IDWI was adopted to determine values at unobserved points in the experiment. The core idea of IDWI is based on the assumption that the attribute value of an unsampled point is weighted related to the values of its neighborhood, and the weights are inversely related to the distances between the predicted location and the sampled locations. Assume the sampled point P_*n*_ = {*x*_*n*_, *y*_*n*_, *v*_*n*_}, where *x*_*n*_ and *y*_*n*_ are its location information and *v*_*n*_ is its attribute value. For the required interpolation point P = {*x*, *y*, *v*}, if *x* and *y* are is known, *v* can be calculated using Equations (1)–(3).
123

**Figure 8:**
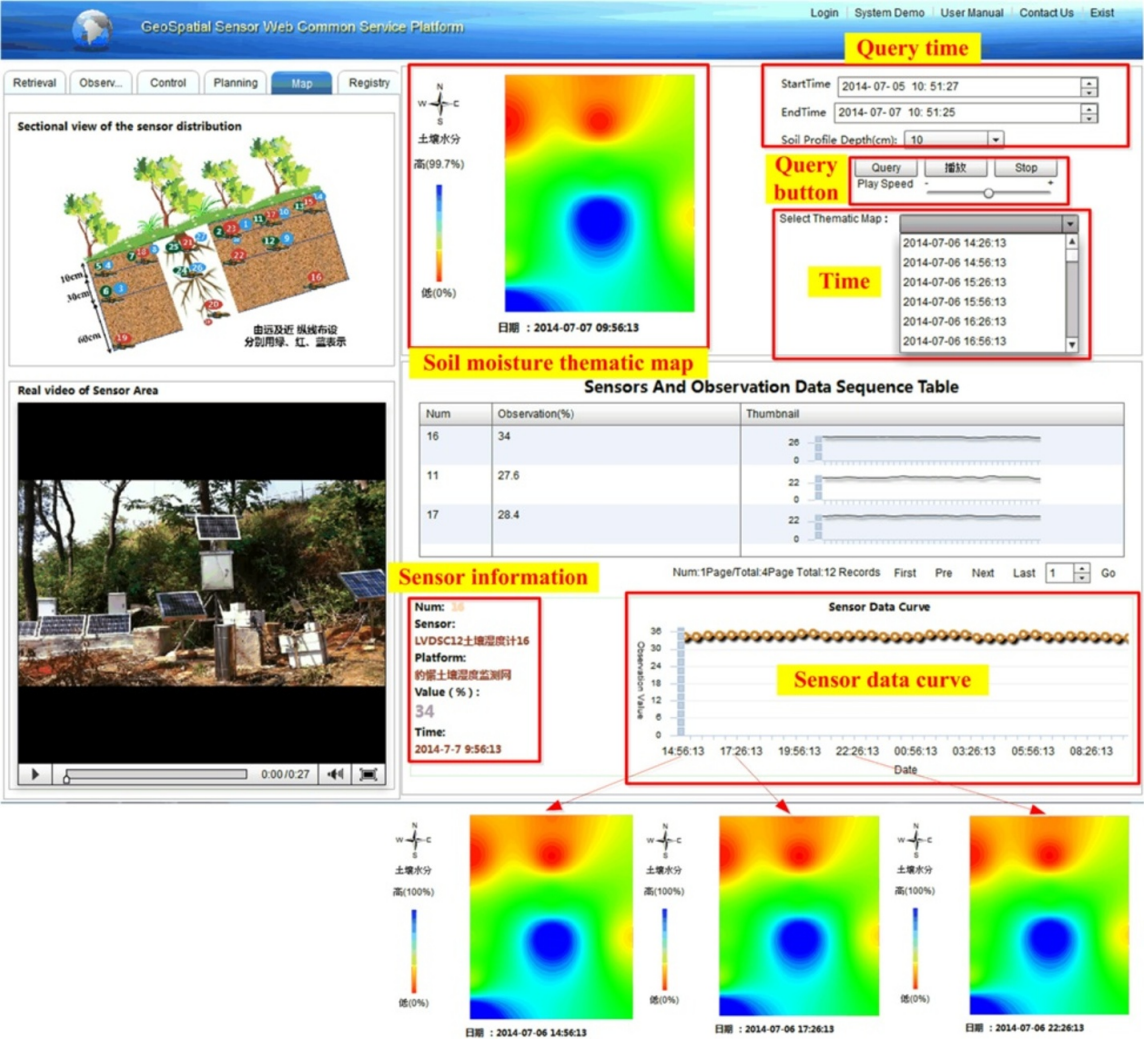
**Soil moisture mapping based on real-time observation data.**

Integrating the Sensor Web Service Platform and the real-time GIS data model as in Table 2, the soil moisture thematic map is realized online using the IDWI method, as shown in Figure 8. The soil moisture mapping is queried during the period from 2014-07-05 10:51:27 to 2014-07-07 10:51:25, as an example. The sensors’ observations at each time point contribute to a thematic map, shown as the soil moisture thematic map at 2014-07-07 09:56:13 in Figure 8. Meanwhile, sensor observation values during the time period contribute to a sensor data curve, as shown in Figure 8. From the soil moisture thematic map, the drought degree in the area can be found. For example, soil moisture conditions in this test area are not balanced in the soil moisture thematic map at 2014-07-07 09:56:13 because the north and the northwest of the area are drought, while the center and the southwest parts of the area are relatively wet. The mapping time of per map is 7.5 s.

## Discussion

We proposed a method based on a novel real-time GIS data model and its realization called the Sensor Web Service Platform for real-time environmental data management. The model, the implementation, and the experiments reflect the real-time characteristic.

The real-time GIS model follows the Geospatial Service Web framework managing all types of geospatial resources from sensor to data, then to information, and finally to knowledge, with the process of obtaining, storing, and processing real-time data. The real-time GIS model is evolved from the temporal GIS model, but it places more emphasis on the time efficiency, having strict time restraints wherein all tasks must be performed in a very short amount of time. The temporal GIS model is always applied to record history data and their changes, while the real-time GIS model points to live data and concerns the current data. The process and causes associated with such real-time data as sensors, geo-objects, states, events, spatiotemporal processes, and functions are considered in the real-time GIS model.

The Sensor Web has real-time or near real-time characteristics [32–35]. Use of the Sensor Web technologies facilitates the realization of real-time characteristics of the real-time GIS model.

In the two experiments, the proposed model and platform manage real-time environmental data. The air quality monitoring sensors, the soil moisture monitoring sensors, and their real-time observations are described with SensorML managed by the SOS and SPS. The sensors are in-situ sensors whose location is fixed during the observation. SensorML can describe both in-situ and mobile sensors [37] such as the GPS receiver sensor on moving taxis in the Sensor Web Service Platform [42] and a camera sensor on a car [35]. The Sensor Web Service Platform obtains and provides real-time observation data using a standard service (see Figures 5 and 7). The two services monitor sensor resources and data resources, while the real-time GIS data model combines the observation and data processes to mine information and knowledge using the real-time data (see Figures 5 and 8). The air quality information from the pollutants is mined using the AQI method, while the soil moisture thematic map is constructed from the observed data using the IDWI method. The time from data collection to observation to server is 1.7 s. The request and visualization time of SOS is 2.0 s. The soil moisture mapping time is 7.5 s. Therefore, the total time efficiency is less than 10 s (1.7 + 7.5 = 9.2, 1.7 + 2.0 = 3.7). The time efficiency means it can meet the requirements of many types of environmental data applications, as the examples (air quality monitoring and soil moisture monitoring) in this study show. The two experiments show the application of the real-time GIS data model and Sensor Web Service Platform, and also use real-time data. Therefore, the real-time GIS data model and Sensor Web Service Platform are seamlessly integrated to manage real-time environmental data.

## Conclusions

The main aim of this study was to propose a method integrating a real-time GIS data model and a Sensor Web Service Platform under a Geospatial Service Web framework for environmental data management. Two experiments, real-time air quality monitoring and real-time soil moisture monitoring in Wuhan, were performed. The experimental results show that using the proposed method to manage real-time environmental data is feasible and effective.

Future work will focus on analyzing the scientific problems associated with the two experimental results. As the objective of the experiments is to demonstrate the proposed model and platform under a GSW framework and their applications for environmental data management, the management processes are illustrated in two experiments, instead of an analysis of the implied meaning and reason for the results.
